# Sound-Evoked Biceps Myogenic Potentials Reflect Asymmetric Vestibular Drive to Spastic Muscles in Chronic Hemiparetic Stroke Survivors

**DOI:** 10.3389/fnhum.2017.00535

**Published:** 2017-11-10

**Authors:** Derek M. Miller, William Z. Rymer

**Affiliations:** ^1^Single Motor Unit Laboratory, Sensory Motor Performance Program, Rehabilitation Institute of Chicago, Chicago, IL, United States; ^2^Interdepartmental Neurosciences Program, Northwestern University, Evanston, IL, United States

**Keywords:** spastic hypertonia, vestibular evoked myogenic potential, stroke, vestibulospinal, motoneuron, vestibular reflexes, biceps brachii

## Abstract

Aberrant vestibular nuclear function is proposed to be a principle driver of limb muscle spasticity after stroke. We sought to determine whether altered cortical modulation of descending vestibulospinal pathways post-stroke could impact the excitability of biceps brachii motoneurons. Twelve chronic hemispheric stroke survivors aged 46–68 years were enrolled. Sound evoked biceps myogenic potentials (SEBMPs) were recorded from the spastic and contralateral biceps muscles using surface EMG electrodes. We assessed the impact of descending vestibulospinal pathways on biceps muscle activity and evaluated the relationship between vestibular function and the severity of spasticity. Spastic SEBMP responses were recorded in 11/12 subjects. Almost 60% of stroke subjects showed evoked responses solely on the spastic side. These data strongly support the idea that vestibular drive is asymmetrically distributed to biceps motoneuron pools in hemiparetic spastic stroke survivors. This abnormal vestibular drive is very likely to be a factor mediating the striking differences in motoneuron excitability between the clinically affected and clinically spared sides. This study extends our previous observations on vestibular nuclear changes following hemispheric stroke and potentially sheds light on the underlying mechanisms of post-stroke spasticity.

## Introduction

**Figure 1:**
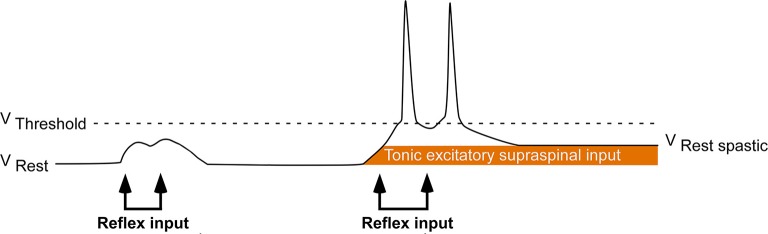
Central hypothesis. Abnormal excitatory ionotropic drive from descending supraspinal pathways perches the baseline membrane potential of contralesional (clinically affected) motoneurons closer to activation threshold, resulting in the lateralized enhancement of stretch reflex excitability.

Following a hemispheric stroke, individuals frequently develop a sharply lateralized increase in muscle tone coupled with augmented tonic stretch reflexes and exaggerated tendon jerks. This combination of signs is called spastic hypertonia or spasticity (Lance, 1980; Urban et al., 2010; Wissel et al., 2013). Spasticity appears to be a delayed consequence of the stroke-mediated interruption of inhibitory corticobulbar fibers that modulate the excitability of brainstem circuitry (Burke, 1988). Indirect evidence suggests that following stroke, spinal motoneurons located on the clinically affected side are placed in a hyperexcitable state. This state is likely the result of a tonic excitatory input that renders these motoneurons more depolarized and thus closer to their activation threshold (Figure 1; Burke and Ashby, 1972; Burke et al., 1972; Powers et al., 1988; Katz and Rymer, 1989; Burne et al., 2005; Mottram et al., 2009; Hu et al., 2015). Such an increase in depolarizing synaptic input could arise from either descending supraspinal pathways or segmental pathways.

In a recent study, we provided evidence indicating that there is an enhancement of sacculocollic drive to the clinically affected sternocleidomastoid motoneuron pool relative to the clinically spared side (Miller et al., 2014). In a subsequent study, we further showed that ascending vestibular drive to the clinically affected and spared extraocular motoneuron pools is also asymmetrically distributed secondary to stroke (Miller et al., 2016). However, it is unknown if descending vestibulospinal drive to cervical motoneuron pools that innervate upper limb muscles is also asymmetrically distributed following a hemispheric stroke, or if the observed asymmetries are unique to the sacculocollic and otolith-ocular pathways. This study summarizes our efforts to quantify the relative levels of vestibulospinal drive to cervical motoneuron pools innervating upper extremity muscles in chronic hemiparetic spastic stroke survivors, and to assess its relationship with spasticity severity.

Vestibular pathway dysfunction has long been suggested to be a principle driver of the increased stretch reflex activity that occurs following a hemispheric stroke (Denny-Brown, 1964, 1965; Burke, 1988; Katz and Rymer, 1989). Decerebrate rigidity is a rough analog of spastic hypertonia, and it occurs following transection of the brainstem between the red nucleus and the vestibular nuclear complex. It is characterized by rigid extension of the limbs and hyperactive stretch reflexes, and is likely driven by unopposed vestibulospinal drive to the motoneuron pools that innervate antigravity musculature (Fulton et al., 1930; Bach and Magoun, 1947). The antigravity limb posturing that follows a cerebral lesion is modified through postural changes and is abolished following transection of the vestibulocochlear nerve (Denny-Brown, 1964, 1965), supporting a potential contributing role for vestibulospinal projections.

As changes in spinal motoneuron excitability after stroke may be at least partly due to alterations in vestibulospinal drive, the aim of the current study was to quantify the relative levels of vestibulospinal drive to the spastic-paretic and contralateral cervical motoneuron pools in chronic stroke survivors presenting with lateralized spasticity secondary to hemispheric stroke. We hypothesized that there would be distinct asymmetries in the amplitudes of acoustically evoked vestibular-mediated reflex responses elicited in the spastic-paretic and contralateral biceps brachii. To test our hypothesis, we recorded sound evoked biceps myogenic potentials (SEBMPs), a stimulus-triggered waveform average recorded from the voluntarily pre-activated biceps brachii in response to intense acoustic stimulation (Luxon, 2013). High-intensity acoustic stimulation is a potent vestibular stimulus (McCue and Guinan, 1994, 1997; Murofushi et al., 1995; Murofushi and Curthoys, 1997). Modulation of EMG in response to acoustic or galvanic vestibular stimulation has been recorded from the recorded from the sternocleidomastoid muscles (cervical vestibular evoked myogenic potential) (Colebatch and Halmagyi, 1992; Colebatch et al., 1994), the inferior oblique eye muscles (Todd et al., 2007; Weber et al., 2012; Miller et al., 2016) as well as in posturally engaged upper limb (Baldissera et al., 1990; Britton et al., 1993; Cherchi et al., 2009), and lower limb musculature (Fitzpatrick et al., 1994; Welgampola and Colebatch, 2001; Bacsi et al., 2003; Rudisill and Hain, 2008). The degree of asymmetry in SEBMP amplitude between the two sides was used as a surrogate measure representing the relative amount of vestibular drive impinging onto the spastic-paretic and contralateral motoneuron pools.

We propose that the stroke-mediated disruption of inhibitory corticobulbar projections causes an imbalance in descending excitatory vestibular drive to motoneurons innervating the antigravity muscles on the spastic-paretic side of patients with lateralized spastic hypertonia. This increased vestibulospinal drive from the contralesional lateral vestibular nuclei is hypothesized to drive baseline membrane potential of spastic-paretic motoneurons closer to their activation threshold, resulting in the lateralized enhancement of stretch reflex excitability, a finding consistent with recent experimental research (Powers et al., 1988; Mottram et al., 2009, 2010; Miller et al., 2014, 2016; Hu et al., 2015).

## Subjects and methods

The musculature on the spastic-paretic side will be classified as clinically affected (CA) and clinically spared (CS) on the contralateral side. As the primary vestibular nuclei involved in regulating the excitability of limb muscles are the lateral vestibular nuclei (VN), the lateral VN on the spastic-paretic side will be referred to as the contralesional VN, while the lateral VN on the contralateral side will be referred to as the ipsilesional VN.

### Subject population

Twelve chronic stroke survivors aged 46–68 years (55.8 ± 6.31 years) who had sustained a cortical lesion resulting in spastic hemiparesis (121 ± 108 months' post-stroke; range, 37–327 months post-stroke) were recruited. In accordance with the Declaration of Helsinki, all subjects gave informed written consent prior to experimentation, and the Northwestern University Institutional Review Board approved all experimental procedures. Subjects reported a negative history for neurological, orthopedic, vestibular, and audiological dysfunction preceding stroke onset. Subjects with severe contracture of the upper extremity muscles, and also associated orthopedic disturbances including shoulder subluxation or shoulder pain were excluded from the study. Hearing acuity was assessed at 250, 500, 1,000, and 2,000 Hz using the Modified Hughson-Westlake paradigm (Earscan 3 Manual Audiometer, Micro Audiometrics Corp., Murphy, North Carolina, USA) to exclude significant hearing loss. Subjects were excluded if they had greater than a 10-dB difference between the left and right ears at 500 Hz. Also, at the time of the study, no subject was taking any vestibular suppressant medications.

Clinical assessments for each subject were performed by a dedicated research physical therapist. Spasticity was assessed at the elbow flexors and the ankle plantar flexors using the Modified Ashworth (MAS) and Modified Tardieu (MTS) scales. The MAS is a 6-point rating scale that is used to measure the resistance of an initially passive muscle (Bohannon and Smith, 1987) and is regarded as the clinical gold standard. The MTS also assesses spasticity; however, it has the advantage over the MAS of separating neural contributions to increased resistance from changes in soft tissue stiffness (Patrick and Ada, 2006). For each subject, we calculated an antigravity spasticity index (AGSI) by dividing the sum of the MAS in the elbow flexors and plantar flexors by eight; the maximum possible attainable MAS score (Miller et al., 2014). Phasic reflex excitability in the biceps (C5-C6) was measured using the deep tendon reflexes. The lower boundary for the tendon jerk was set at a score of 2+. Functional capacity was assessed in the upper extremities using the 66-point upper extremity motor domain of the Fugl-Meyer Assessment, where a higher score is indicative of higher motor function. Table 1 details demographic and clinical information for each subject. Notably, we excluded four subjects; two subjects based on having no spasticity in the upper or the lower limbs (MAS 0) and two subjects that had hypoactive deep tendon reflexes.

**Table 1:** Subject demographic and clinical information.

**Subject ID:**	**Age**	**Sex**	**MPS**	**Lesion location**	**Side of Paresis/Hand Dominance**	**MAS EF**	**MAS PF**	**AGSI**	**UEFM**
**1. BIL (*n* = 4 subjects)**									
5	57	M	301	U	L/R	2	1	0.38	20
12	68	M	124	R periatrial WM–microhemorrhage L medial temporal lobe	L/R	2	0	0.25	15
14	50	M	41	L BG/posterior IC/centrum semi-ovale	R/L	0	1	0.13	41
18	54	F	60	L MCA hemorrhage	R/R	0	1	0.13	49
**2. USPA (*n* = 7 subjects)**									
3	49	M	45	R IC	L/R	1	1	0.25	42
8	64	M	248	L MCA	R/R	3	0	0.38	8
13	46	F	37	R IC/thalamic/corona radiata	L/R	1+	3	0.56	19
17	52	F	37	L BG hemorrhage	R/R	1	1	0.25	10
20	58	M	95	L BG or lateral thalamic	R/R	2	1	0.38	17
21	54	M	59	L thalamic hemorrhage	R/L	1+	1+	0.38	16
22	60	F	327	L IC/BG	R/R	1+	1+	0.38	4
**3. UCON (*n* = 1 subject)**									
16	57	M	77	R BG (PR)	L/R	1	1	0.25	18

U, unknown; WM, white matter; BG, basal ganglia; IC, internal capsule; PR, patient-reported; MCA, middle cerebral artery.

### Data acquisition

**Figure 2:**
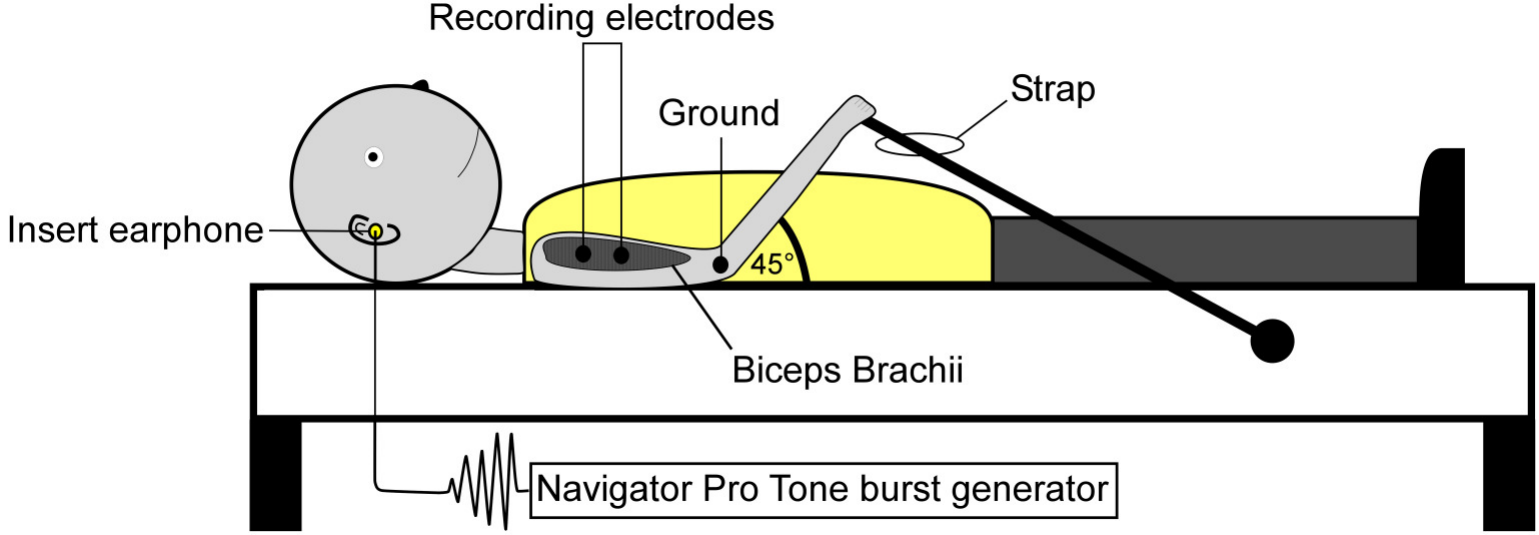
Subject positioning and electrode montage used for the collection of SEBMPs. SEBMPs were collected from the tonically active biceps brachii muscles in 12 chronic stroke subjects. Surface electrodes were placed distally on the biceps muscle belly, a third of the distance between the medial acromion and the fossa cubit (interelectrode distance, 25–30 mm). A ground electrode was placed over the epicondyle process. Subjects lay supine on an examination table and were instructed to maintain a moderate isometric contraction in the biceps brachii by pulling against a strap attached to the examination table.

Surface electromyographic (EMG) recordings were collected from both the CA and CS biceps brachii. Prior to electrode placement, the skin was prepared using isopropyl alcohol and abrasive prep tape (3M Red Dot Trace Prep, 3M Corp., St. Paul, Minnesota, USA). Self-adhesive disposable Ag/AgCl electrodes were placed distally on the biceps muscle belly (NeuroPlus 22.2 × 34.9 mm solid gel electrodes, Vermed Inc., Bellows Falls, Vermont, USA), a third of the distance between the medial acromion and the fossa cubit (interelectrode difference 25–30 mm). Depending on the subject, a ground electrode was placed on the lateral or medial epicondyle process of the humerus. Figure 2 illustrates subject positioning and electrode placement. EMG signals were amplified x500, band-pass filtered 0.3–1 kHz (Model LP-511 High Performance AC Amplifier, Grass Technologies, West Warwick, Rhode Island, USA) and digitally sampled at 5 kHz using a 1401 analog-to-digital converter (625 kHz 16-bit Power 1401) linked to a PC running Spike 2 version 7.10 software (Cambridge Electrode Design, Cambridge, UK).

### Acoustic stimuli

Acoustic stimuli were generated using the Biologic Navigator Pro Auditory Evoked Potential system (Natus Medical, San Carlos, California, USA). Short tone bursts were delivered binaurally at an impulse intensity of 85 dB nHL (120 SPL) through standard foam E-A-RTone 3A insert earphones (E-A-R Auditory Systems, Indianapolis, Indiana, USA). Each 500 Hz tone burst was presented at 4 per second with rarefaction polarity and Blackman ramping (5 ms plateau; 1 ms rise and fall). Each trial consisted of two consecutive trains of 128 tone bursts each and yielded a stimulus-triggered waveform average of 256 repetitions sampled over a 200 ms epoch (50 ms prior to 150 ms after stimulus onset).

### Experimental procedures

Experiments were conducted in a quiet, dimly lit room. Visual input was excluded by instructing the subject to keep his or her eyes closed. Subjects lay supine on an examination table and were instructed to maintain a moderate isometric contraction in the CA or CS biceps brachii by pulling against a strap that was attached to the examination table (Figure 2). Background EMG activity was monitored online using a custom-designed software program (MATLAB and Statistics Toolbox Release 2012b, The Mathworks, Inc., Natick, Massachusetts, USA). Verbal feedback was used to inform the subject if they strayed from a predetermined level of preactivation. Preactivation levels typically ranged from 10 to 40% (±10%) maximal voluntary contraction or MVC. However, the ability to match a particular percent MVC depended on the level of impairment or the recording conditions. To establish biceps brachii preactivation target levels, subjects performed at least two MVCs. The MVC raw data were rectified, and a 3-s window was used to estimate the mean activity level.

A randomly presented sham trial consisting of no acoustic stimulation was used to verify that responses were not due to inherent variability in the baseline EMG activity or to signal processing methods. A random number generator determined the presentation order of the trials (*left or right biceps; sound or sham paradigm*). In addition to the sham trial, between two and five active trials were obtained from each side. The number of trials depended on the reproducibility and consistency of the response. Testing occurred in one session that lasted approximately 3 h. However, in almost every case, the subject was brought in for a follow-up visit. Intertrain (60–120 s) and intertrial (300–420 s) rest periods minimized muscle fatigue. Data sets were analyzed offline using custom-designed software programs (Spike 2; MATLAB and Statistics Toolbox Release 2012b).

### Waveform analysis

**Figure 3:**
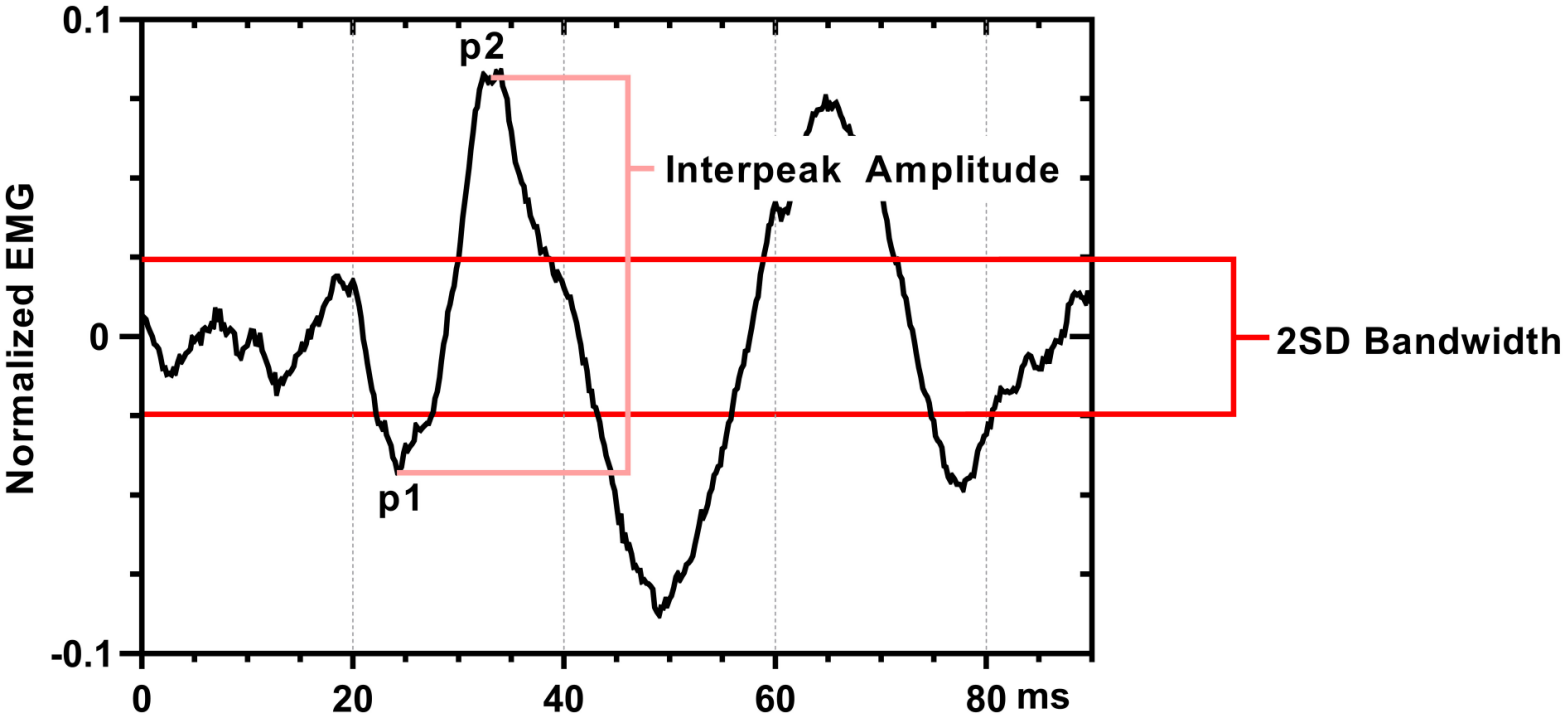
SEBMP waveform analysis. The SEBMP waveform is a stimulus-triggered average generated in response to binaural acoustic stimulation. A peak was labeled p1 if it occurred between 20 and 75 ms and exceeded two standard deviations above baseline based on the prestimulus unrectified EMG (50 ms period). The subsequent peak of opposite polarity immediate following p1 was designated p2. The interpeak amplitude and interpeak interval were calculated from the unrectified waveform average.

We defined a positive SEBMP response as a reproducible biphasic waveform with an interpeak amplitude that exceeded a two-standard deviation bandwidth, calculated from the unrectified prestimulus waveform average over a 50 ms epoch. The CA and CS SEBMP waveform for each side represented the average of two consistent runs with stable EMG activation levels and were free from noise (Figure 3). A peak was labeled p1 if it occurred between 20 and 75 ms post-stimulus onset and exceeded the two-standard deviation bandwidth. The subsequent peak of opposite polarity was designated as p2. Peak onset latencies were calculated with respect to stimulus onset and were defined as p1 and p2. The p1p2 interpeak amplitude was calculated by summing the absolute magnitudes of the p1 and p2 peaks. Interpeak intervals were calculated by subtracting the p1 peak onset latencies from the p2 peak onset latencies. The peak onset latencies (ms), p1p2 interpeak amplitude (μV) and the interpeak interval (ms) were measured from the unrectified waveform average. Responses not distinguishable from background noise or that were not repeatable were considered absent.

Interpeak amplitudes were normalized to the mean prestimulus (50 ms) rectified EMG (corrected interpeak amplitude, dimensionless). Normalization with respect to prestimulus EMG activity allows for differences in preactivation levels, as the amplitude of the myogenic potential is known to scale with the level of background activity (Colebatch et al., 1994). Side-to-side differences in vestibulospinal drive were quantified by expressing the CA and CS corrected interpeak amplitudes as an asymmetry ratio (AR, %). The magnitude of the corrected interpeak amplitude and asymmetry ratio was used as a measure of the relative levels of descending vestibulospinal drive impinging upon the CA and CS motoneuron pools. For subjects that had no response on either the CA or CS side, the response amplitude was marked as zero (0) and calculated as a 100% asymmetry.

$$\begin{array}{l} Asymmetry\;Ratio\;\left(AR\right)=\\ 100^{*}\frac{\left(CA\;Interpeak\;Amplitude-CS\;Interpeak\;Amplitude\right)}{\left(CA\;Interpeak\;Amplitude+CS\;Interpeak\;Amplitude\right)} \end{array}$$

### Statistical analysis

All data are expressed in terms of mean ± standard deviation unless otherwise noted. A *p*-value ≤ 0.05 was considered significant. Statistical analyses were performed using Prism version 6.00 for Windows (Graph Pad Software, La Jolla, California, USA). The corrected interpeak amplitude, peak onset latencies, and interpeak interval from each subject were averaged to calculate the CA and CS population means. A paired two-tailed Student's *t*-test was used to analyze any significant differences in the CA and CS corrected interpeak amplitudes. Pearson correlation analysis was used to calculate the strength of the relationship between the CA corrected interpeak amplitude and the antigravity spasticity index. Based on their response profile, subjects fell into one of three categories: (1) BIL subjects had responses present bilaterally on both the CA and CS sides; (2) USPA subjects had responses present on the CA side; and (3) UCON subjects' responses were present on the CS side only. Population means, standard deviations, ranges, and 95% confidence intervals (CI) are detailed in the text below.

## Results

### BIL subjects

Four subjects (33%) had SEBMPs present bilaterally, on both the CA and CS sides. These four subjects ranged in age from 50 to 68 years (57.3 ± 7.7 years) and were 131.5 ± 118.4 months' post-stroke (41 to 301 months). Our major finding here is that the magnitude of the interpeak amplitude was 30% larger on the CA than that on the CS side. The mean corrected interpeak amplitude was 0.42 ± 0.16 (range, 0.24–0.62; 95% CI, 0.16–0.68) on the CA side and 0.30 ± 0.07 (range, 0.23–0.40; 95% CI, 0.19–0.42) on the CS side. The mean AR (%) was 13.5 ± 19.1, ranging from minus 7 to 33.

Mean rectified EMG background activity was somewhat smaller on the affected side: 26.9 ± 12.1 μV (CA) and 40.7 ± 7.8 μV (CS). The mean CA p1 peak onset latency was 53.0 ± 2.9 ms (range, 49.0–56.0: 95% CI, 48.3–57.6). When compared to the CA side, the mean p1 peak onset latency on the CS side was approximately 13% shorter, 46.3 ± 12.3 ms (range, 30.6–60.4; 95% CI, 26.8–65.8). The p2 peak onset latency was 64.7 ± 5.2 ms (range, 59.8–71.4; 95% CI, 56.5–72.9) and 55.0 ± 13.2 ms (range, 40.2–72.4; 95% CI, 33.9–76.0) on the CA and CS sides respectively (16% difference). Responses were on average 29% longer in interpeak duration on the CA side: 11.7 ± 3.0 ms (CA) versus 8.7 ± 3.0 ms (CS).

### USPA subjects

**Figure 4:**
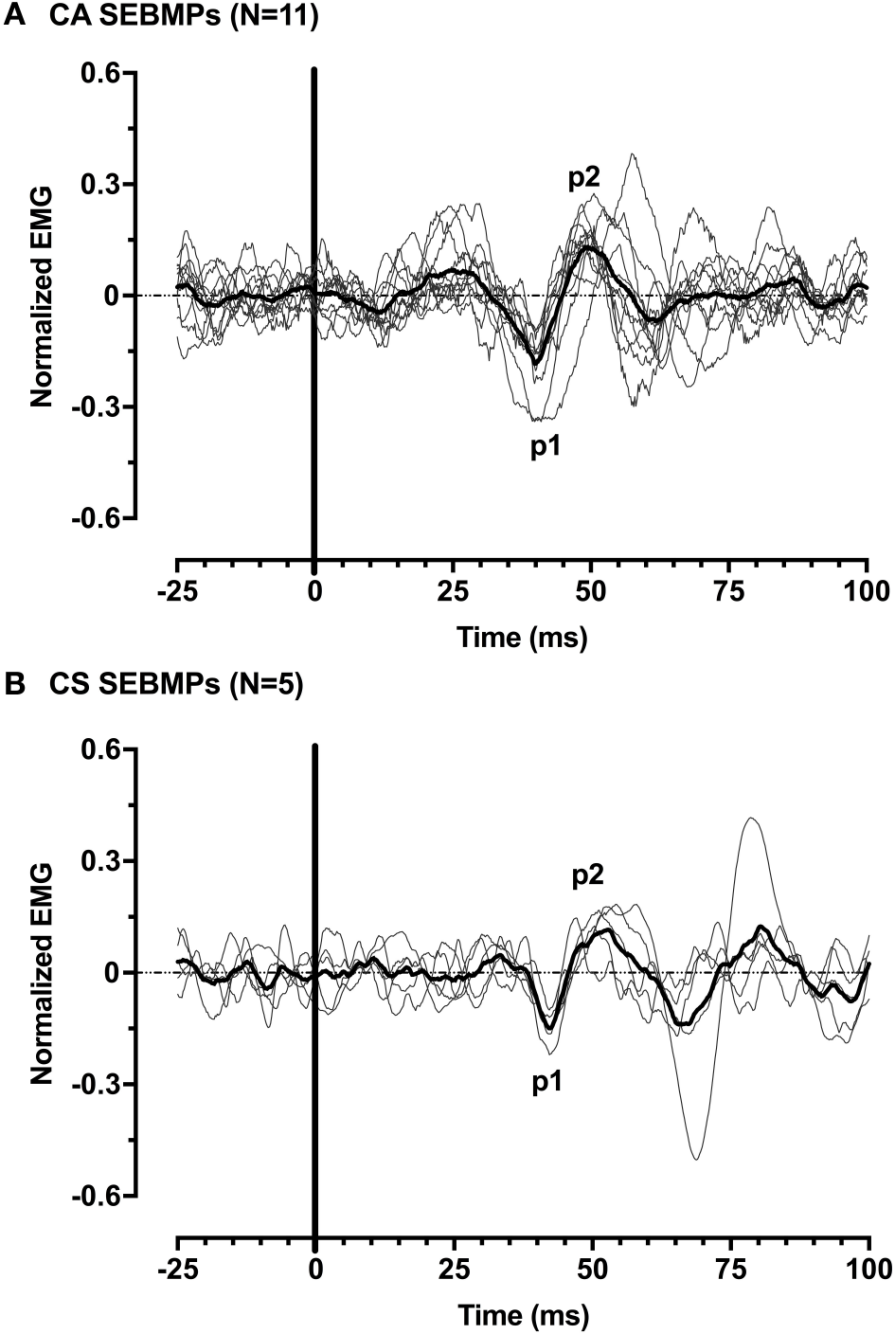
Normalized SEBMPs from the CA **(A)** and CS **(B)** biceps brachii muscles of chronic stroke subjects. The CA and CS population averages are superimposed (thick line) over the individual responses. For clarity, the individual responses are normalized with respect to p1 (latency).

A second major finding here was that in 7/12 (58%) subjects, *consistent responses were present only on the CA side*. The mean age for the USPA group was 55 ± 6 years, ranging from 46 to 64 years. Subjects averaged 121 ± 118 months post-stroke (range, 37–327 months) and had mild to moderate spasticity. Functionally, subjects in this group ranged from mild to severe (FM Score 4–42; mean, 16.6 ± 12.4). Five of the seven subjects had sustained left-sided lesions while the remaining two had right-sided infarctions that involved the internal capsule. The mean CA corrected interpeak amplitude was 0.39 ± 0.16 (range, 0.24–0.72; 95% CI: 0.24–0.53). Overall, mean rectified EMG activity was smaller on the affected side: 17.3 ± 5.8 μV (CA) and 54.6 ± 34.7 μV (CS). Compared to the subjects' in-group BIL, p1 [32.4 ± 9.1 ms (range, 22.2–47.0; 95% CI, 24.0–40.8)] and p2 [42.6 ± 11.3 ms (range, 31.6–64.6; 95% CI, 32.1–53.1)] peak onsets were decreased. However, the responses showed similar interpeak interval duration (10.2 ± 3.5 ms). Figure 4 shows the individual and averaged normalized SEBMPs from the CA and CS sides of group BIL and UPSA subjects.

**Figure 5:**
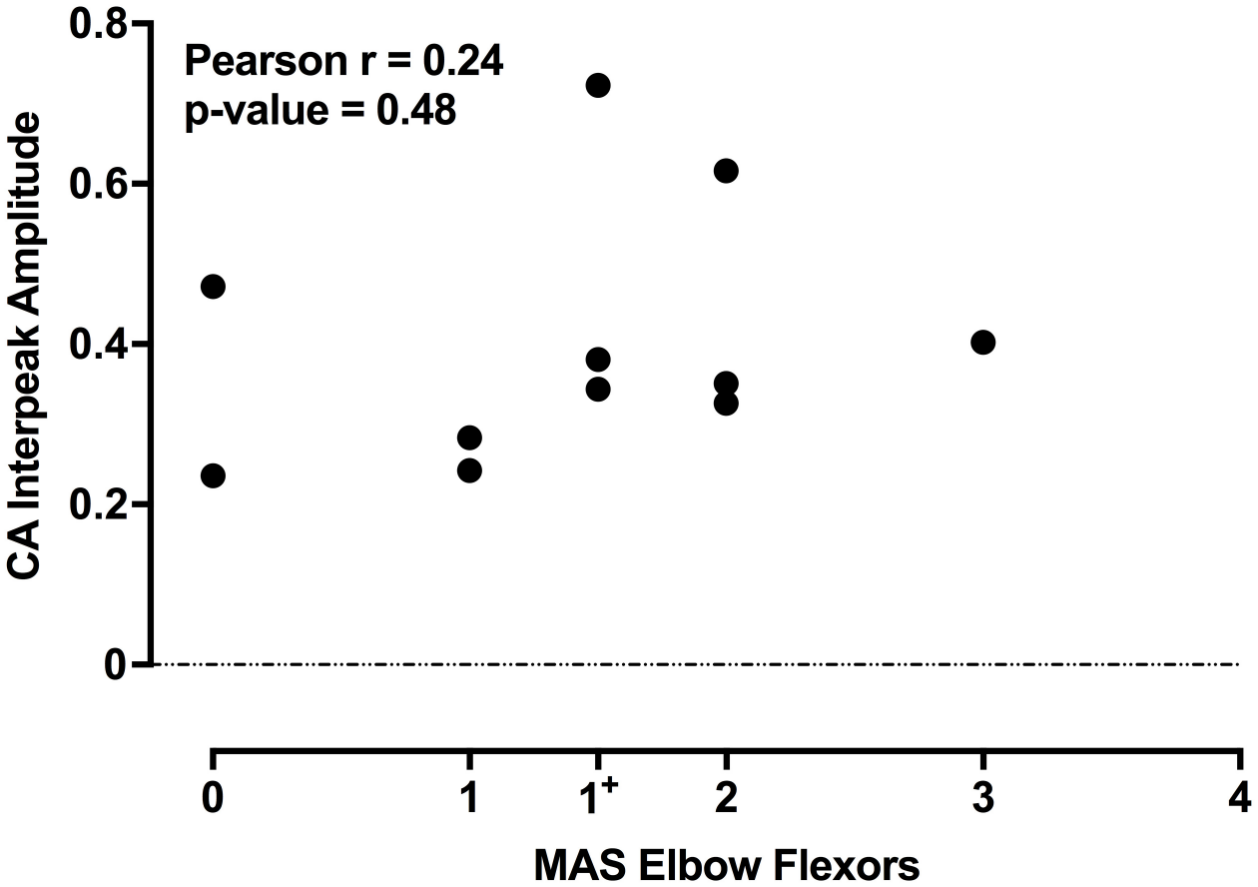
For the 11 subjects in groups BIL and USPA, there is a weak-positive, but a non-significant relationship between the CA corrected interpeak amplitude and the severity of spasticity in the elbow flexors. The coefficient of determination and *p*-value are indicated in the upper left of the figure.

**Figure 6:**
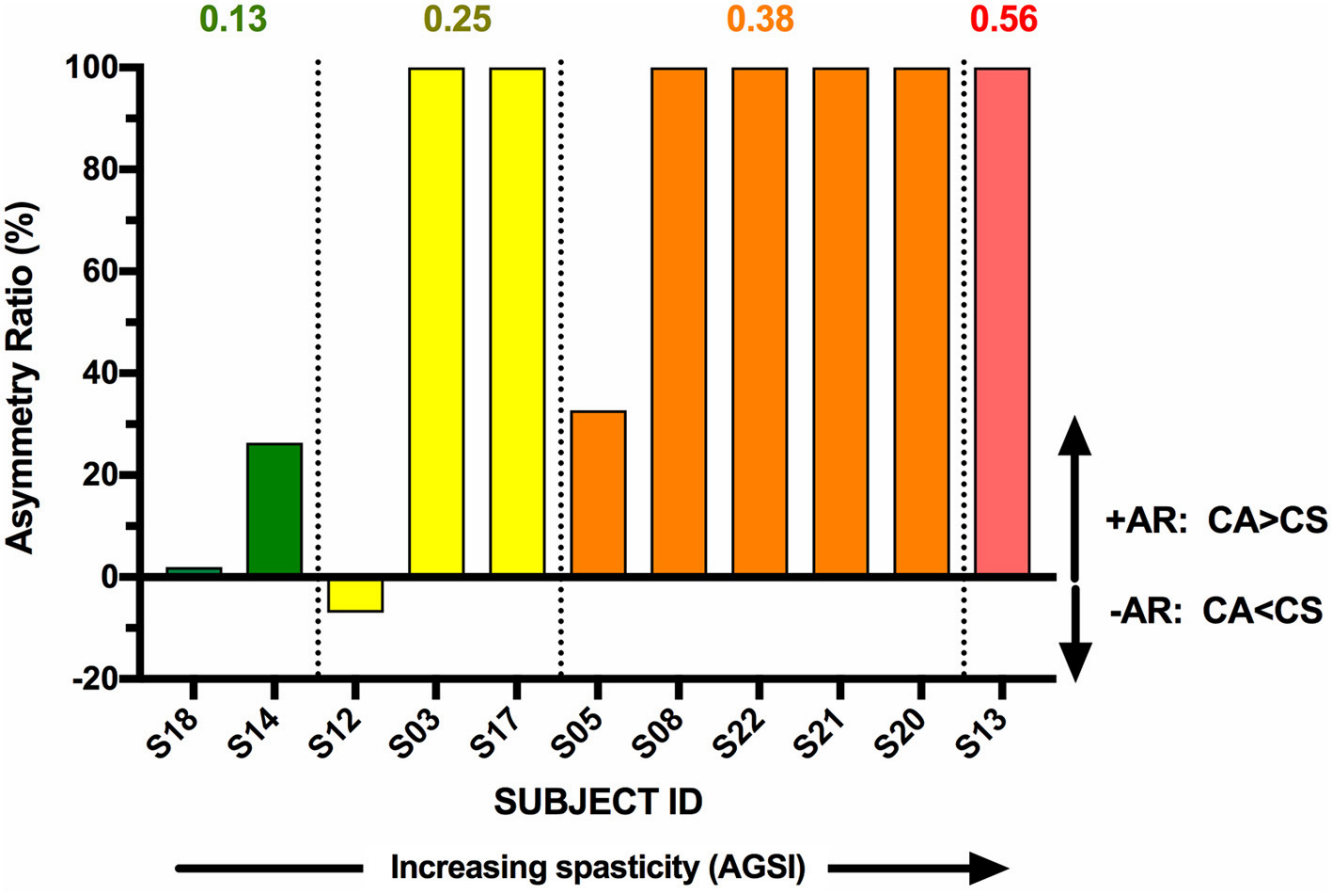
BIL and USPA subjects were binned per the severity of spasticity, determined by the antigravity spasticity index (AGSI). Within each bin, subjects were rank-ordered in terms of increasing AR.

The individual CA corrected interpeak amplitudes from the 11 subjects (group BIL and USPA) were plotted as a function of the Modified Ashworth Score in the elbow flexors (biceps). There was a weak positive but non-significant relationship between CA response amplitude and the severity of spasticity in the biceps (Figure 5; *n* = 11 subjects; Pearson *r* = 0.24, *p* = 0.48). To further explore the relationship between CA response amplitude and the severity of spasticity, we plotted the CA corrected interpeak amplitudes from the 11 subjects in group BIL and USPA as a function of the antigravity spasticity index. There was a weak positive relationship between the magnitude of the interpeak amplitude and the severity of spasticity; however, it was not significant (*n* = 11 subjects; Pearson *r* = 0.26, *p* = 0.43). Individual asymmetry ratios for the 11 BIL and USPA subjects were binned into groups based on the AGSI ratio affirming the striking asymmetry of the responses between CA and CS sides (Figure 6). When all three groups are combined, the mean corrected interpeak amplitudes for the CA and CS sides were 0.365 ± 0.185 (range: 0–0.723; 95% confidence interval [CI]: 0.247–0.482) and 0.129 ± 0.164 (range: 0–0.404; 95% CI: 0.025–0.243), respectively. The CA corrected interpeak amplitude was significantly larger than the CS corrected interpeak amplitude (*n* = 12 subjects, 2-tailed paired *t*-test, *p* = 0.0104).

### UCON subjects

One subject (16) was a 57-year-old right-handed male that had sustained an ischemic infarct in the area of the right basal ganglia (PPR) 77 months prior to study enrollment. The subject was moderately impaired (FM score, 18) and had minimal spasticity that was equally distributed between the upper and lower limbs. The CS corrected interpeak amplitude was 0.3, with p1 and p2 peak onset latencies of 26.0 ms and 41.8 ms respectively. Background EMG values were 17.7 μV (CA) and 101.8 μV (CS).

## Discussion

The striking differences in muscle tone that exist between the alert but quiescent animal and the decerebrate preparation highlight the powerful influence that the cortex has over descending supraspinal pathways. The loss of cortical influence over brainstem output pathways after the interruption of presumptive inhibitory corticobulbar fibers is believed to result in a net disinhibition of brainstem nuclei (Magoun and Rhines, 1946), and it is likely that this loss of cortical modulation of brainstem nuclei is a mechanism common to both hemispheric stroke and decerebration injuries. Although not a precise analog for human stroke, the loss of descending corticobulbar pathways following intercollicular transection parallels many of the changes in muscle tone and posture that occur secondary to stroke involving the posterior limb of the internal capsule in humans. Direct evidence for corticobulbar projections from the premotor and vestibular cortices to the contralateral caudal medial and lateral VN in primates has been demonstrated using retrograde tracers injected into the vestibular nuclear complex (Akbarian et al., 1993, 1994). These corticobulbar projections could descend in proximity to the corticospinal tract (Terao et al., 2000; Marsden et al., 2005) or they could arise as collaterals of corticospinal axons (Keizer and Kuypers, 1984; Marsden et al., 2005). We do not yet know which is the most likely scenario.

**Figure 7:**
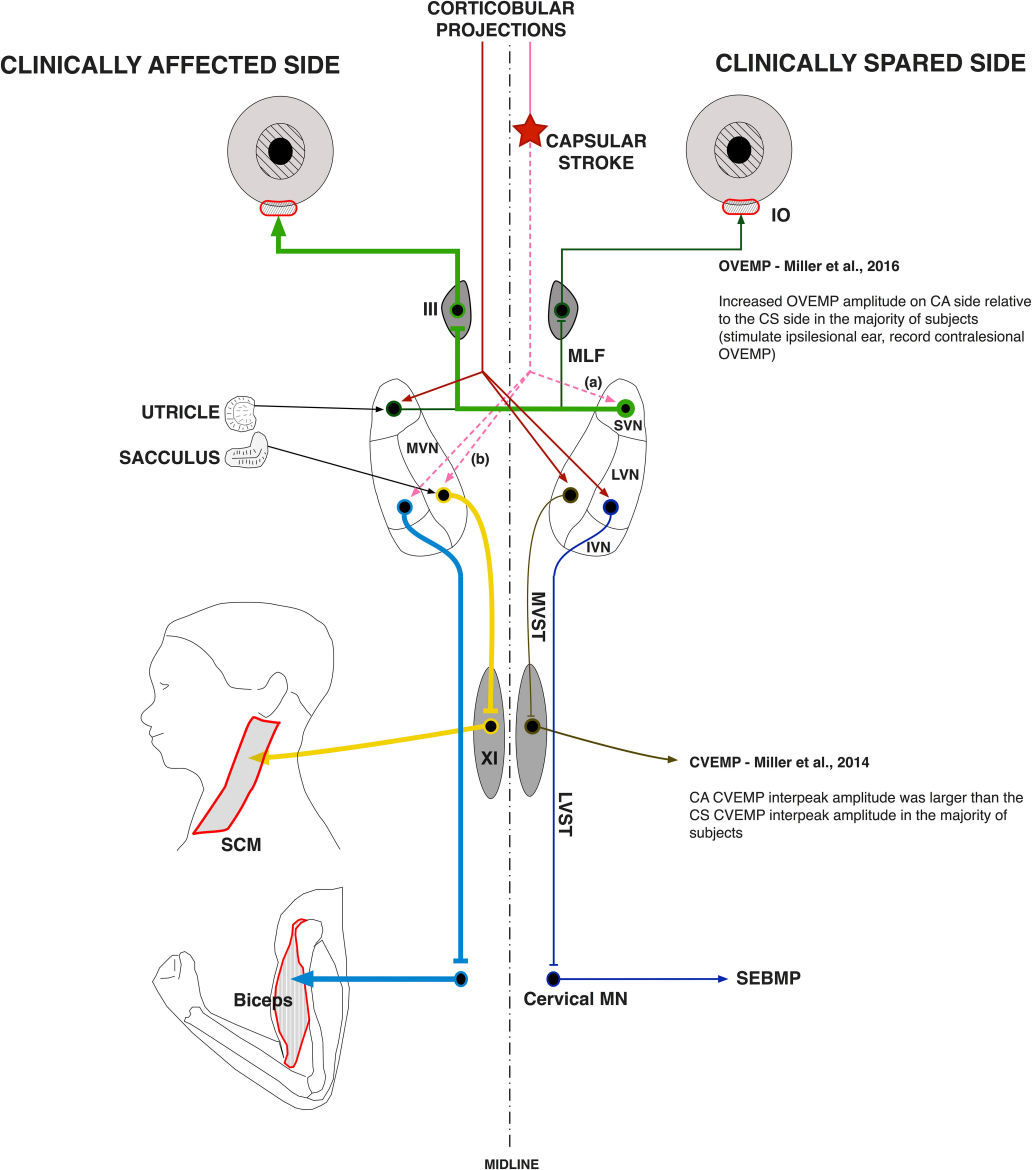
Vestibular pathway abnormalities post-stroke. The cerebral cortex exerts powerful control over the vestibular nuclear complex. It is linked to the contralateral MVN and LVN and the ipsilateral SVN by fiber pathways originating in the premotor and vestibular cortices. Our main hypothesis is that the lateralized disruption of corticobulbar projections causes an imbalance in descending vestibular drive to the spinal motoneuron pools, which sets the membrane potential of spastic motoneurons closer to their activation threshold. Here, a lateralized disruption (star) in corticobulbar projections (dashed lines) releases the (a) ipsilateral SVN and the (b) contralateral LVN and MVN as well as their associated ascending and descending pathways from inhibitory cortical control (thick lines). The thickness of the pathway lines represents relative levels of activity within the given pathway. The results of the current study support a lateralized disruption in vestibular pathways post-stroke, especially when added to our previous work.

Our observations that vestibular evoked EMG responses are greatly enhanced in hemispheric stroke survivors fit well with this model of disinhibition of brainstem nuclei by the stroke-induced interruption of corticobulbar projections (Figure 7). Indeed, almost 60% of our stroke subjects exhibited evoked responses *solely* on the clinically affected side. These data strongly support the idea that vestibular drive is asymmetrically distributed to biceps motoneuron pools in spastic stroke survivors. This abnormal vestibular drive is thus very likely to be a major factor mediating the striking differences in motoneuron excitability between the clinically affected and clinically spared sides.

It is plausible that the lack of responses on the clinically spared side in the USPA group is the result of extensive intrinsic connections that functionally link the VN on one side (Rubertone et al., 1983; Epema et al., 1988) and potent inhibitory commissural pathways that interconnect the left and right VN in a push-pull configuration (Carleton and Carpenter, 1983; Buttner-Ennever, 1992; Furuya et al., 1992). Therefore, an increase in activity on one side results in a simultaneous decrease in activity on the other side. It follows that a stroke-mediated increase in contralesional VN activity would also indirectly cause decreased activity in the ipsilesional VN via commissural pathways, and of the ipsilesional lateral VN through intrinsic connections. Potentially, this could help explain our results, whereby with increasing spasticity, neurons located in the contralesional lateral VN and medial VN are increasingly disinhibited, driving them closer to their activation threshold. In parallel, there is a subsequent decrease in activity in the ipsilesional lateral VN and medial VN.

SEBMP waveforms are morphologically similar to acoustically-evoked myogenic responses previously measured from the biceps brachii (Luxon, 2013), triceps brachii (Cherchi et al., 2009) and the gastrocnemius muscles (Rudisill and Hain, 2008). Given the known orientation of the electrode coupled with the excitatory actions of lateral vestibulospinal pathways, the p1-peak likely represents the synchronous activation of the motoneuron pool (Figure 3). Moreover, peak response latencies are comparable to those reported in the upper limb muscles, ranging from 30 to 50 ms (Baldissera et al., 1990; Britton et al., 1993; Cherchi et al., 2009; Luxon, 2013). The range of onset latencies is likely a dual reflection of the use of different stimulation modalities (Bacsi et al., 2003) and the known task dependency of vestibulospinal reflexes (Fitzpatrick et al., 1994). The SEBMP response is likely mediated via a polysynaptic pathway arising in the lateral vestibular nucleus and descends via the lateral vestibulospinal tract. Direct monosynaptic connections, while present, are rare relative to indirect connections that are mediated through interneuronal elements (Nyberg-Hansen and Mascitti, 1964; Akaike, 1983). Furthermore, it is known through studies in the cat that most vestibulospinal neurons terminate on interneurons rather than directly on the motoneuron (Nyberg-Hansen, 1964).

The observed lack of correlation between SEBMP interpeak amplitude and the severity of spasticity could reflect the use of binaural stimulation and/or the multimodal nature of the vestibulospinal reflexes. In the current study, we used binaural as opposed to monaural acoustic stimulation to elicit SEBMPs. While monaural stimulation was attempted, but we could not reliably evoke responses in the biceps muscle. Therefore, it is likely that the response is generated through the activation of both sets of primary afferents and the input is integrated within the vestibular nuclear complex before the descending modulatory command is sent to motoneuron pool. As the response is apt to be the consequence of both ipsi- and contralesional VN activity, it follows that we would not expect to see strong correlations between reflex amplitude and the severity of spasticity.

Another possible reason for the lack of correlation is that vestibular mediated reflex responses in postural muscles are modulated by multimodal sensory information from vestibular, visual, somatic, and proprioceptive inputs. While visual inputs were eliminated, proprioceptive inputs as well as somatosensory inputs, could have differed between the spastic-paretic and contralateral limbs. For example, while every attempt to control the amount of elbow flexion was made, more impaired individuals with significant spasticity often had difficulty with the isometric force generation task due to a lack of mobility and a decreased range of motion on the spastic-paretic side. Differing somatosensory and proprioceptive inputs from joint capsules and muscles could potentially confound our results.

It is well-known that loud sounds, such as those utilized in the SEBMP and VEMP protocols, can trigger a startle response. The classic acoustic startle reflex is a subcortical reflex that is characterized by widespread EMG responses elicited in many muscle groups (Brown et al., 1991). The classic startle reflex evoked in the biceps brachii has an onset latency of roughly 69 ms, and while a response can be seen in as few as one stimulus presentation, the response amplitude typically habituates within 2–6 trials (Brown et al., 1991). We do not believe that the startle response plays a significant role here for two reasons. First, we matched excitability of motoneurons closely on both sides of our stroke survivors, yet our SEBMP responses remain heavily lateralized. Second, when compared to startle evoked biceps responses in control and stroke subjects (Jankelowitz and Colebatch, 2004), the peak onset latencies were significantly shorter across all subjects. Given these lines of converging evidence, it does not appear that the responses we measured were of startle origin.

While an uncompensated increase in descending vestibulospinal drive is the most parsimonious explanation for our findings, alternative explanations are conceivable. Spastic-paretic motoneuron hyperexcitability could result from an increase in the intrinsic excitability of the motoneuron itself through the activation of dendritic L-type Ca^2+^ and TTX-sensitive persistent Na^+^ channels, both of which engage voltage-sensitive channels that are subject to robust modulation by descending monoaminergic drive (Hounsgaard et al., 1988). Augmented persistent inward currents would increase the likelihood of motoneuron discharge through the amplification and prolongation of synaptic input (Lee and Heckman, 2000). However, there is sparse and inconsistent evidence for the enhancement of persistent inward currents in spasticity of cerebral origin (Mottram et al., 2009, 2010). Additionally, the bilateral and diffuse nature of brainstem monoaminergic projections (Björklund and Skagerberg, 1982) does not readily explain the sharply lateralized nature of stroke-induced spasticity. Alternatively, changes in segmental reflex transmission may occur following stroke. It is conceivable that a disruption of descending pathways that mediate presynaptic inhibition could contribute to our findings. The resulting depression of presynaptic inhibition would increase the efficacy of input from the 1a afferents in firing the motoneuron (Pierrot-Desseilligny and Burke, 2005). While there is evidence for a post-stroke reduction in presynaptic inhibition, it is again bilaterally distributed within the spinal cord, occurring on both the spastic-paretic and contralateral sides, and it correlates poorly with the severity of spasticity (Lamy et al., 2009). It also has been suggested that increased fusimotor drive contributes to reflex abnormalities. However, there is no evidence for a post-stroke enhancement of spindle afferent sensitivity (Wilson et al., 1999). It is likely that secondary to stroke there are changes at the level of the supraspinal nuclei and the segmental reflex arc with the extent likely depending on the site and scope of the hemispheric lesion.

Previous research shows a lateralized increase in startle reflex amplitude in about 25% of stroke subjects (Jankelowitz and Colebatch, 2004). Cortical involvement is questioned, due in part to the finding that spinal cord injured patients also exhibited increased startle, indicating a potential change at the segmental level (Jankelowitz and Colebatch, 2004). Additionally, cortico-reticulospinal pathways cannot easily explain the sharply lateralized nature of spasticity due to their inherent anatomy. Projections from the premotor cortex to the pontomedullary reticular formation are bilateral (Matsuyama and Drew, 1997; Rho et al., 1997), and the reticulospinal tracts are mostly bilateral in both their anatomical spinal distribution (Nyberg-Hansen, 1965) and synaptic action (Schepens and Drew, 2006; Davidson et al., 2007). However, involvement of reticular pathways cannot be entirely dismissed given the extensive interconnections between the vestibular and reticular complexes (Ladpli and Brodal, 1968; Carleton and Carpenter, 1983).

In summary, our current observations suggest that there is an asymmetric distribution of vestibulospinal drive to cervical motoneuron pools in a subset of spastic stroke survivors. We propose that asymmetric vestibular input is a source of an uncompensated low-level ionotropic drive to the motoneuron pool that keeps spinal motoneurons on the spastic-paretic side closer to their activation threshold and thus more readily activated by afferent-mediated synaptic input. However, more research is needed to evaluate the effect of vestibular drive on spinal motoneuron excitability post-stroke.

## Author contributions

Conception or design of the work: WR and DM; Data collection: DM; Data analysis and interpretation: DM and WR; Drafting the article: DM; Critical revision of the article: WR and DM; Final approval of the version to be published: WR and DM.

### Conflict of interest statement

The authors declare that the research was conducted in the absence of any commercial or financial relationships that could be construed as a potential conflict of interest.
